# Dysregulation of the tumor suppressor Menin and its target Bach2 in HTLV-1 infection

**DOI:** 10.1186/s12977-025-00660-7

**Published:** 2025-03-25

**Authors:** Hiroe Sejima, Tadasuke Naito, Takuya Fukushima, Mineki Saito

**Affiliations:** 1 Department of Microbiology, Kawasaki Medical School, 577 Matsushima, Kurashiki, Okayama 701-0192 Japan; 2 Laboratory of Hematoimmnology, School of Health Sciences, Faculty of Medicine, University of the Ryukyus, 207 Uehara, Okinawa, 903-0215 Japan

**Keywords:** HTLV-1, Menin, Bach2, HAM/TSP, ATL, Tax, HBZ

## Abstract

**Background:**

The tumor suppressor Menin, prone to mutations in both hereditary and sporadic endocrine tumors, along with its direct target Bach2, plays a crucial role in preventing autoimmunity by regulating CD4 + T cell senescence and maintaining cytokine homeostasis. Since human T-cell leukemia virus type 1 (HTLV-1) primarily infects CD4 + T cells, and its dysregulation contributes to both the hematological malignancy of adult T-cell leukemia/lymphoma (ATL) and HTLV-1-associated myelopathy/tropical spastic paraparesis (HAM/TSP), we examined the involvement of the Menin-Bach2 pathway in HTLV-1 infection.

**Methods:**

The mRNA expression of *menin* and *bach2* in HTLV-1-infected and uninfected human T-cell lines, peripheral blood mononuclear cells (PBMCs) from patients with ATL, HAM/TSP, and asymptomatic carriers were analyzed. Additionally, interactions between Menin or Bach2 and the Tax or HBZ; the subcellular localization of these proteins; the effect of knockdown of *menin*, *tax*, and *HBZ* genes; and the effects of interaction inhibitors between menin and its cofactor, mixed lineage leukemia (MLL), on the proliferation of HTLV-1-infected T cells were evaluated.

**Results:**

The findings were as follows: (1) In all eight HTLV-1-infected T-cell lines tested, Menin protein was expressed, whereas Bach2 expression was absent in five of them; (2) the mRNA levels of both *menin* and *bach2* significantly decreased in PBMCs from patients with HAM/TSP and ATL; (3) Tax and HBZ each physically interacted with both Menin and Bach2; (4) knockdown of *tax*, but not *HBZ*, downregulated Bach2, but not Menin expression in HTLV-1-transformed T-cell lines MT-2 and SLB-1; (5) knockdown of *menin* downregulated Bach2 expression in MT-2 but not in SLB-1; (6) A Menin-MLL interaction inhibitor suppressed cell growth of MT-2 but not in SLB-1; (7) HBZ and Menin exhibited different subcellular localization between MT-2 and SLB-1.

**Conclusions:**

HTLV-1 infection alters the regulation of the Menin-Bach2 pathway, which controls cell proliferation. The Menin-MLL interaction inhibitor loses its effectiveness in suppressing cell proliferation when Menin loses control over Bach2 expression. Dysregulation of the Menin-Bach2 pathway may contribute to HTLV-1-associated disease pathogenesis.

**Supplementary Information:**

The online version contains supplementary material available at 10.1186/s12977-025-00660-7.

## Background

Human T-cell leukemia virus type 1 (HTLV-1) was the first human retrovirus to be identified and has been associated with at least two different forms of disease, including adult T-cell leukemia/lymphoma (ATL) [1–3] and HTLV-1-associated myelopathy/tropical spastic paraparesis (HAM/TSP) [4, 5]. In contrast to HIV infection, only a small percentage of HTLV-1-infected individuals develop the disease after the virus has persisted for an extended period of time, that is, 2–5% for ATL [6] and 0.25–3.8% for HAM/TSP [7–10], while the vast majority remain lifelong asymptomatic carriers (ACs). As HTLV-1 primarily infects CD4 + T helper (Th) cells, which play an important role in the adaptive immune response [11], it has been speculated that HTLV-1-related diseases may be caused by the accumulation of multiple abnormalities in infected individuals during a long incubation period, particularly in CD4 + T cells. However, little is known about why some HTLV-1-infected individuals develop various diseases while most infected individuals remain asymptomatic. In this regard, HTLV-1 infection may be a suitable model to study the pathological mechanisms of malignancy and chronic inflammation, and to develop therapeutic agents and vaccines for these diseases.

Menin is a tumor suppressor protein mutated in patients with multiple endocrine neoplasia type 1 (MEN1), an autosomal dominant disease characterized by parathyroid hyperplasia, pancreatic islet cell tumors, and anterior pituitary endocrine tumors [12]. It is generally believed that Menin dysfunction due to downregulation or mutation is associated with increased cell proliferation [13], and that Menin is required for the proliferation and survival of antigen-stimulated CD4 + T cells in vivo [14]. The transcriptional repressor Bach2 (broad complex-tramtrack-bric a brac and Cap’n’collar homology 2), which was initially discovered as a key player in antibody class switching and somatic hypermutation [15], has been reported as a direct downstream target of Menin [14]. Menin binds to the Bach2 locus and controls its expression by maintaining histone acetylation [14]. Furthermore, Menin binding at the Bach2 locus and subsequent downregulation of Bach2 expression in CD4 + T cells results in an “senescence-associated secretion phenotype (SASP),” which is characterized by high levels of inflammatory cytokines, immunomodulators, growth factors and proteases secreted after antigen stimulation, as well as dysregulated cytokine production [16]. Bach2 plays a critical role in regulating cellular immune responses in vivo by controlling the functions of regulatory T cells (Tregs) [17, 18], CD8 + memory T cells [19], and CD4 + effector T cells [14, 17, 20]. Thus, the Menin-Bach2 pathway is important for age-related immune dysfunction and susceptibility to chronic inflammation and cancer. Based on these findings, we investigated the involvement of the Menin-Bach2 pathway in HTLV-1 infection and related diseases.

## Results

### Loss of Bach2 expression in HTLV-1-infected T cell lines

**Fig. 1:**
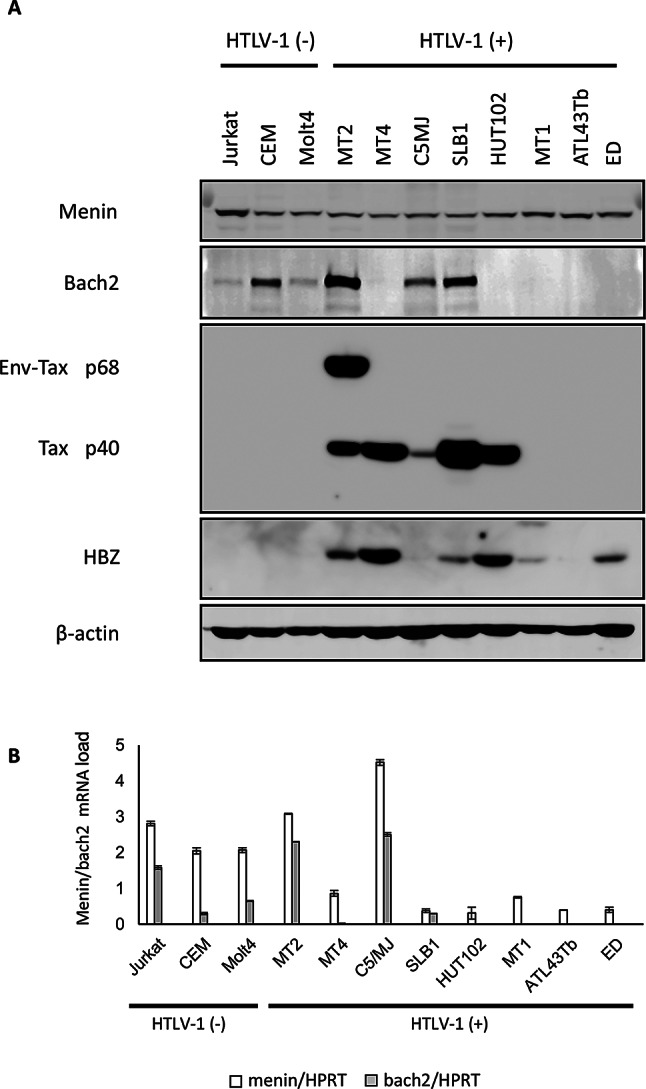
Decreased expression of Bach2 in HTLV-1-infected T-cell lines. (**A**) Expression of Menin, Bach2, Tax, and HBZ proteins was examined by western blot in HTLV-1-infected and -uninfected T-cell lines. (**B**) The expression levels of *menin* and *bach2* mRNA in HTLV-1-infected and -uninfected T-cell lines was examined by qRT-PCR

First, the protein levels of Menin, Bach2, Tax, and HBZ were examined by western blotting in HTLV-1-infected and uninfected T cell lines. As shown in Fig. 1A, Menin was expressed in all T-cell lines, regardless of HTLV-1 infection, that is, HTLV-1-transformed T-cell lines obtained by co-culture of PBMCs or cord blood leukocytes with leukemic T cells from an ATL patient (MT-2, MT-4, C5/MJ, and SLB-1) or ATL patient-derived leukemic T-cell lines (HUT102, MT-1, ATL43Tb, and ED). In contrast, Bach2 was not detected in the majority of HTLV-1-infected T cell lines (was absent in five out of eight) and was expressed in all three HTLV1-negative human leukemia T cell lines tested (Jurkat, CEM, and Molt4), although the expression levels varied. Notably, HTLV-1 infected cell lines expressed Bach2 after 5-aza-2’-deoxycytidine treatment (Supplementary Fig. 1), suggesting that Bach2 is silenced by an epigenetic mechanism. No correlation was found between the Bach2 protein levels and HTLV-1 Tax or HBZ levels in the tested cell lines (Fig. 1A), probably due to translation efficiency, protein stability, or cellular regulatory mechanisms. Next, the expression levels of *menin* and *bach2* mRNA in HTLV-1-infected and -uninfected T-cell lines were examined using qRT-PCR. As shown in Fig. 1B, there was no correlation between the mRNA and protein levels for both genes. To examine whether Bach2 protein expression is induced by the HTLV-1 trans-activator protein Tax, we used JPX9 cells [21], a Jurkat subclone generated by the stable introduction of a functional Tax expression-plasmid vector, and induced Tax expression by adding CdCl_2_ to the culture medium (final concentration: 10 µM). Western blot analysis showed that treatment of JPX9 cells with CdCl_2_ induced Tax expression, and Bach2 expression was augmented after Tax induction, suggesting that Tax is involved in the upregulation of Bach2 in a human leukemic T-cell line (Supplementary Fig. 2).

### Decreased mRNA expression levels of *menin* and *bach2* in PBMCs of HAM/TSP and ATL patients

To confirm whether the in vivo expression profiles of *menin* and *bach2* mRNA were related to the clinical status of HTLV-1 infection, the expression levels of *menin* and *bach2* mRNA in PBMCs from HTLV-1 infected individuals and normal uninfected controls (NCs) were examined by qRT-PCR in 21 patients with ATL (acute-type, *n* = 12; lymphoma-type, *n* = 6; smoldering-type, *n* = 2; chronic-type, *n* = 1) and 13 patients with HAM/TSP, 11 ACs, and 11 NCs.

**Fig. 2:**
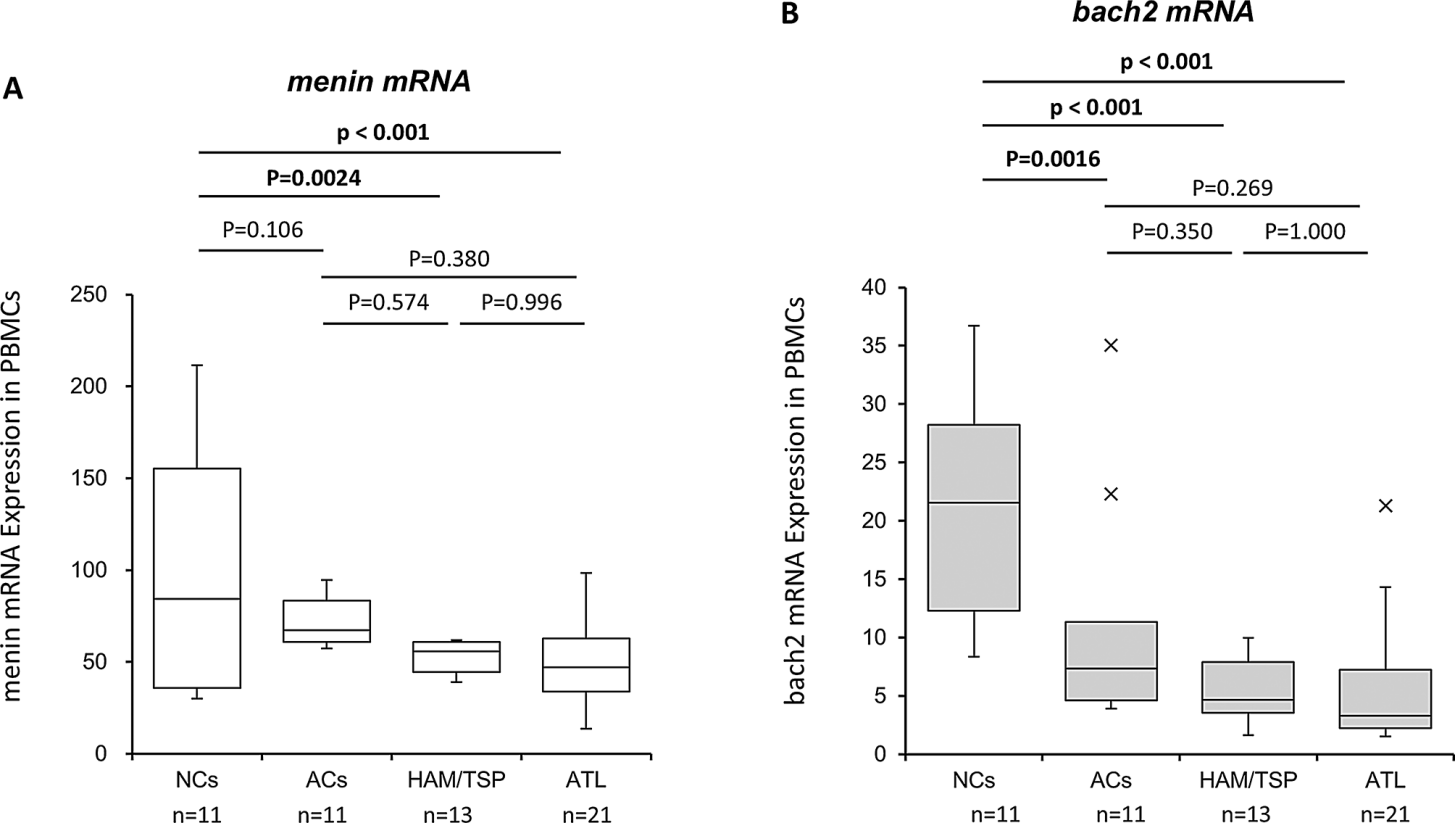
The decline in the mRNA expression levels of *menin* and *bach2* in PBMCs from both HAM/TSP and ATL patients. (**A**) Expression of *menin* and *bach2* mRNA in PBMCs from HTLV-1 infected individuals and normal uninfected controls (NCs) was examined in 21 patients with ATL (acute-type, *n* = 12; lymphoma-type, *n* = 6; smoldering-type, *n* = 2; chronic-type, *n* = 1), 13 patients with HAM/TSP, 11 ACs, and 11 NCs by qRT-PCR. Statistically significant reduction was observed in the expression level of *menin* mRNA in both HAM/TSP and ATL patients than NCs. (**B**) Expression of *bach2* mRNA in PBMCs from HTLV-1 infected individuals and NCs was examined by qRT-PCR. The expression level of *bach2* mRNA in HTLV-1 infected individuals was significantly lower than were in NCs, irrespective of infection status (i.e., HAM/TSP, ATL, ACs)

The results demonstrated a statistically significant reduction in the expression level of *menin* mRNA in both HAM/TSP and ATL patients compared to that in NCs (Fig. 2A), whereas the expression level of *bach2* mRNA in HTLV-1 infected individuals was significantly lower than that in NCs, irrespective of the infection status (i.e., HAM/TSP, ATL, and ACs) (Fig. 2B).

### Tax and HBZ each physically interacts with both Menin and Bach2

**Fig. 3:**
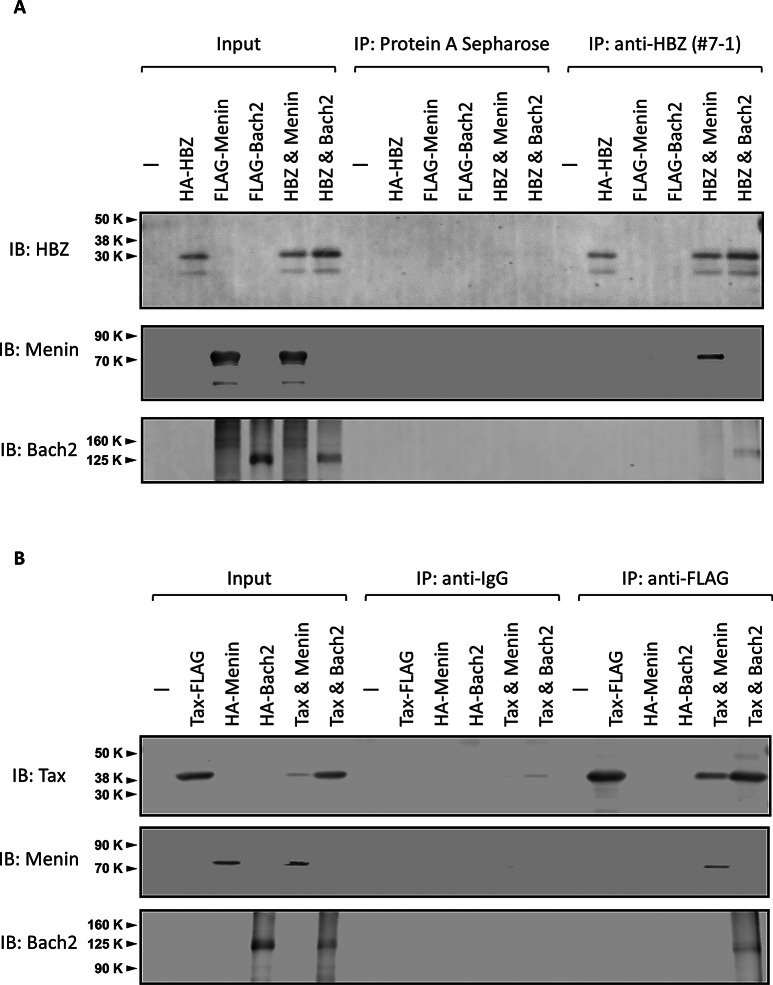
Tax and HBZ each physically interacts with both Menin and Bach2. Physical interactions between Menin-Bach2 pathway members (i.e., Menin or Bach2) and viral regulatory proteins (Tax or HBZ) were investigated using co-immunoprecipitation. (**A**) HBZ physically interacts with Menin and Bach2. (**B**) Tax physically interacts with both Menin and Bach2

Thus, the characteristics of the clinical samples suggest the dysregulation of the Menin-Bach2 pathway in HTLV-1 infection. Therefore, we investigated the physical interactions between Menin-Bach2 pathway members (i.e., Menin or Bach2) and viral regulatory proteins (i.e., Tax or HBZ) by co-immunoprecipitation. Human embryonic kidney (HEK) 293T cells were transfected with each expression plasmid for the co-immunoprecipitation assay. Tax and HBZ physically interacted with both Menin (Fig. 3A) and Bach2 (Fig. 3B). Meanwhile, co-immunoprecipitation with MT-2 cells failed to detect endogenous Bach2 protein (Supplementary Fig. 3).

### Menin-MLL interaction inhibitor have opposing effects on cell proliferation in two HTLV-1-transformed T-cell lines MT-2 and SLB-1

**Fig. 4:**
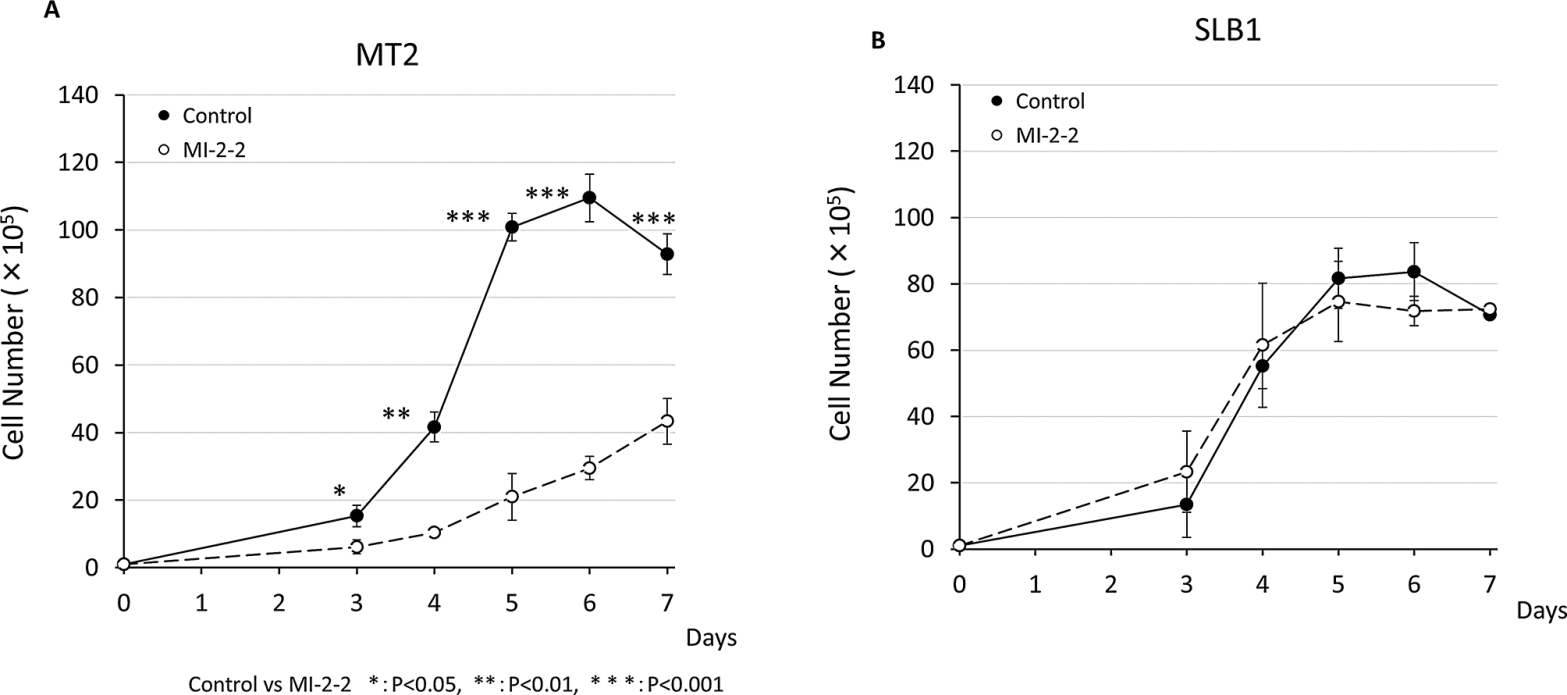
Menin-MLL interaction inhibitor have opposing effects on cell proliferation in two HTLV-1-infected T-cell lines. The effect of the Menin-MLL inhibitor, MI-2-2, on the growth of two types of HTLV-1 infected human T-cell lines, the ATL-derived HTLV-1-infected T-cell line SLB-1 and the HTLV-1-transformed cell line MT-2, was examined. The unfilled, white circle indicates the data with MI-2-2 (12 µM); the filled-in, black circles indicate the data without MI-2-2. (**A**) MI-2-2 inhibited cell proliferation of MT-2. (**B**) MI-2-2 did not inhibit cell proliferation of SLB-1

It has been reported that the Menin functions as a critical oncogenic co-factor of mixed lineage leukemia (MLL) fusion proteins in development of acute leukemia, and inhibition of the Menin-MLL interaction is a promising strategy to reverse their oncogenic activity [22]. Since our present findings suggest that HTLV-1 infection alters the regulation of the Menin-Bach2 pathway, which controls cell proliferation, it is possible that the inhibition of Menin suppresses the proliferation of HTLV-1 infected cells therefore has therapeutic potential against ATL. Moreover, if the Menin inhibitor MI-2-2 shows different effects on the proliferation of various HTLV-1 infected cell lines, comparative analysis of Tax or HBZ kinetics along with Bach2 kinetics may provide new therapeutic targets or points of action for ATL. Therefore, we examined the effect of MI-2-2 [23] on HTLV-1-transformed T-cell lines SLB-1 and MT-2, as these cells express all factors of interest (i.e., Menin, Bach2, Tax, and HBZ) at the protein level. A total of six HTLV-1-infected cell lines (MT1, MT4, HUT102, C5MJ and SLB1) and three HTLV-1-uninfected cell lines (Jurkat, CEM and Molt4) were examined, but only two of the HTLV-1-infected cell lines exhibited growth inhibition in response to MI-2-2 (12 µM) treatment (Supplementary Fig. 4 and Fig. 4).

### Knockdown of HTLV-1 Tax by shRNA results in decrease of Bach2 but not Menin protein in HTLV-1-infected T-cell lines

**Fig. 5:**
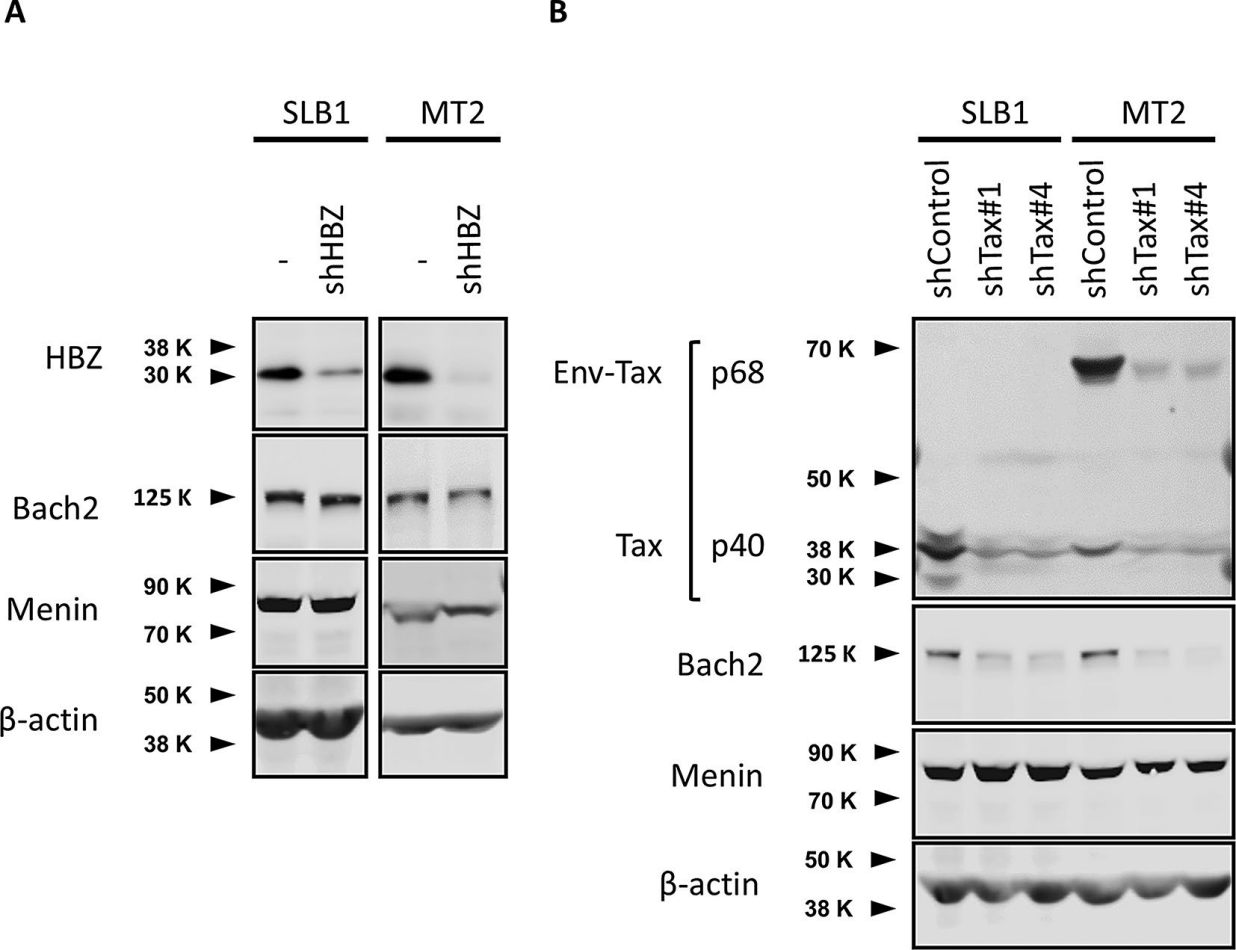
Knockdown of HTLV-1 Tax by shRNA results in decrease of Bach2 but not Menin protein in HTLV-1-infected T-cell lines. Tax- or HBZ-directed shRNAs in HTLV-1-infected T cell lines showed opposite effects on cell growth by the Menin-MLL interaction inhibitor. The shRNA knockdown of HBZ did not change the expression levels of either Menin and Bach2 in SLB-1 and MT-2 cells (**A**), whereas the shRNA knockdown of Tax suppressed the expression levels of Bach2 but not Menin in SLB-1 and MT-2 cells (**B**)

To investigate the functional significance of the viral regulatory proteins Tax and HBZ in two HTLV-1-transformed T-cell lines that show opposite effects on cell growth, we transfected SLB-1 and MT-2 cells with lentiviral vectors expressing Tax- or HBZ-directed small hairpin RNAs (shRNAs). As shown in Fig. 5A, shRNA knockdown of HBZ did not change the expression levels of either Menin and Bach2 proteins in SLB-1 and MT-2 cells. In contrast, shRNA knockdown of Tax suppressed the expression levels of Bach2 but not Menin, in SLB-1 and MT-2 cells (Fig. 5B).

### Knockdown of Menin by siRNA results in decrease of Bach2 in HTLV-1-infected T-cell line MT-2, in which the cell growth is suppressed by Menin-MLL interaction inhibitor

**Fig. 6:**
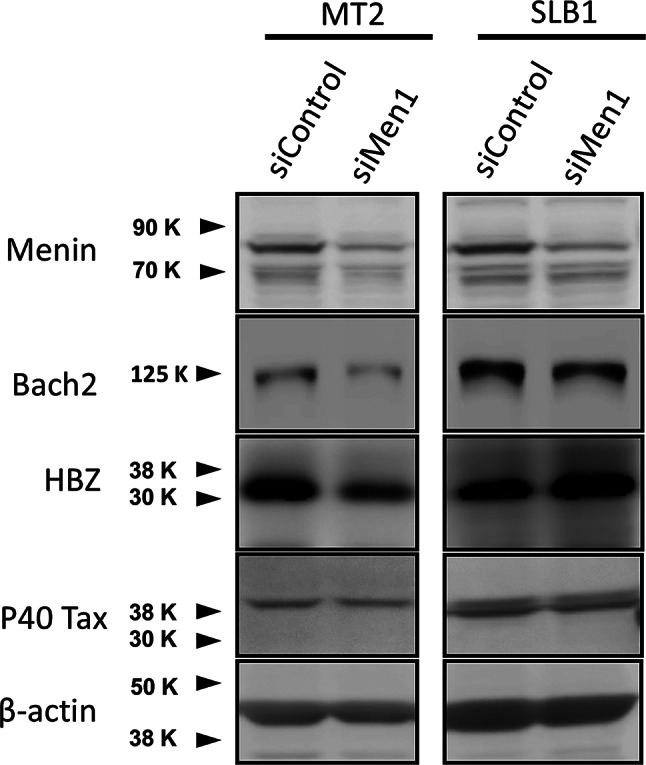
Knockdown of Menin by siRNA results in decrease of Bach2 in HTLV-1-infected T-cell line MT-2, in which the cell growth is suppressed by Menin-MLL interaction inhibitor. The effect of *menin*-directed siRNAs in HTLV-1-infected T cell lines showed opposite effects on cell growth to the Menin-MLL interaction inhibitor. The siRNA knockdown of Menin resulted in a decrease of Bach2 in the HTLV-1-infected T-cell line MT-2, in which cell growth was suppressed by the Menin-MLL inhibitor MI-2-2, whereas siRNA knockdown of Menin did not change the expression levels of Bach2 protein in SLB-1 cells, in which cell growth was not affected by the Menin-MLL inhibitor MI-2-2

To investigate the functional significance of the Menin-Bach2 pathway in two HTLV-1 transformed T cell lines that showed opposite effects on cell proliferation by Menin-MLL interaction inhibitor MI-2-2, small interfering RNAs (siRNAs) were used to suppress *menin* gene expression. As shown in Fig. 6, the siRNA-mediated knockdown of Menin resulted in a decrease in Bach2 in MT-2 cells, in which cell growth was suppressed by MI-2-2 (see Fig. 4A). The knockdown of Menin did not change the expression levels of Bach2 protein in SLB-1 cells, in which cell growth was not affected by MI-2-2 (Figs. 4B and 6).

### HBZ and Menin exhibited different subcellular localization between MT-2 and SLB-1 cells

**Fig. 7:**
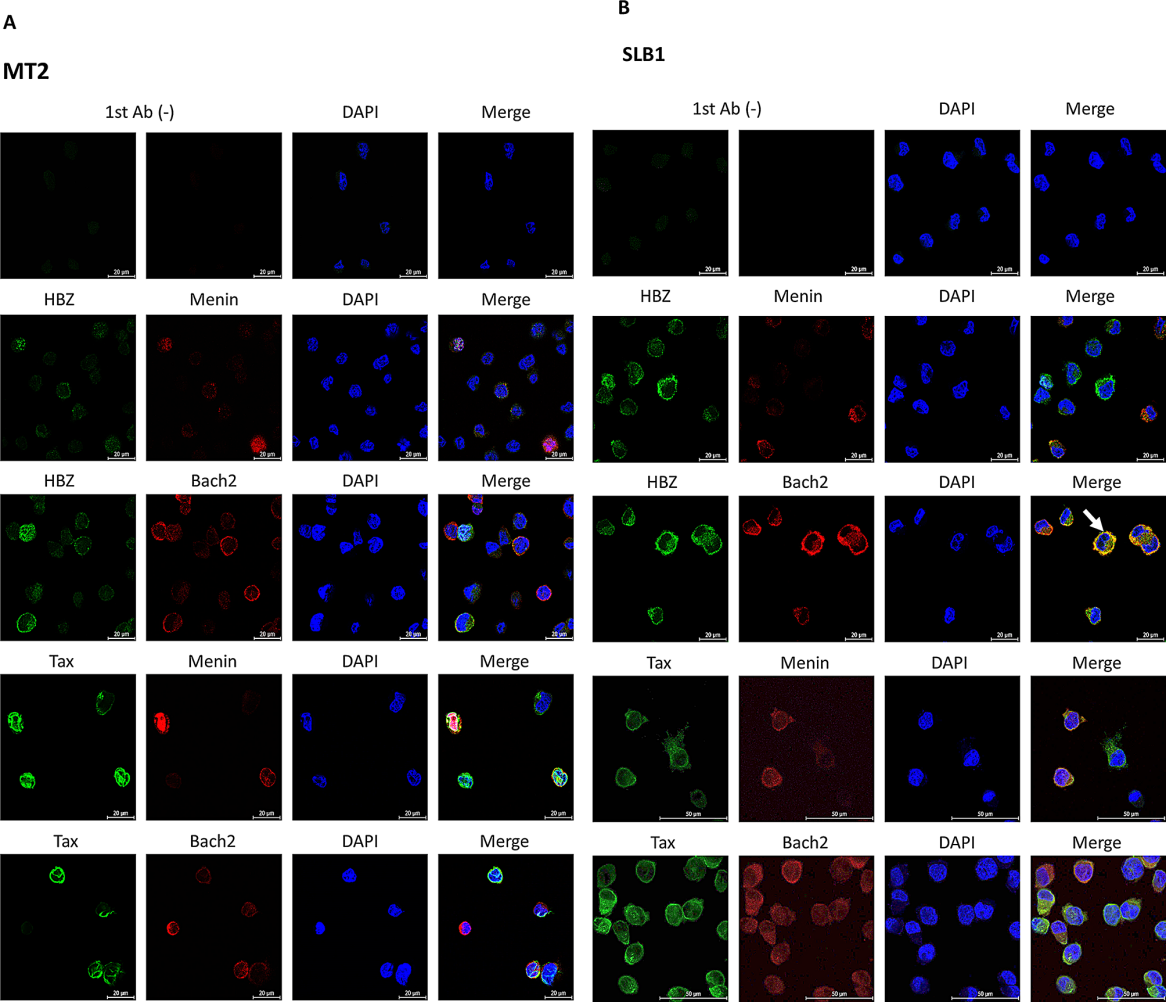
HBZ and Menin exhibited different subcellular localization between MT-2 and SLB-1, which shows opposite effects on cell proliferation by Menin-MLL interaction inhibitor. The subcellular localization of Menin and Bach2 in SLB-1 and MT-2 cells was determined and compared with that of the HTLV-1 regulatory factors Tax and HBZ by indirect immunofluorescence staining. Tax was localized in the cytoplasm of both MT-2 and SLB-1 cells, whereas HBZ showed different localization patterns in both cell lines. (**A**) HBZ was predominantly localized in the nucleus of MT-2 cells. Menin was mainly localized in the nuclei of MT-2 cells. Bach2 is localized in the cytoplasm of MT-2 cells. (**B**) HBZ is predominantly localized in the cytoplasm of SLB-1 cells. Menin was predominantly localized in the cytoplasm of SLB-1 cells. Bach2 is localized in the cytoplasm of SLB-1 cells. HBZ and Bach2 were partially co-localized in the cytoplasm of SLB-1 cells (white arrow)

To further investigate the significance of the Menin-Bach2 pathway in HTLV-1 transformed T cell lines, which showed opposite effects on cell proliferation by inhibitors of the Menin-MLL interaction inhibitor MI-2-2, the subcellular localization of Menin and Bach2 in SLB-1 and MT-2 cells was determined and compared with the subcellular localization of the HTLV-1 regulatory factors Tax and HBZ by indirect immunofluorescence staining (Fig. 7A and B, and Supplementary Fig. 5A). Regarding viral regulatory factors, Tax is localized in the cytoplasm of both MT-2 and SLB-1 cells. In contrast, HBZ was localized predominantly in the nucleus of MT-2 cells (Fig. 7A) and predominantly in the cytoplasm of SLB-1 cells (Fig. 7B). Regarding cellular factors, Menin was localized mainly in the nucleus of MT-2 cells (Fig. 7A) but was predominantly localized in the cytoplasm of SLB-1 cells (Fig. 7B). Bach2 was localized in the cytoplasm of both MT-2 and SLB-1 cells (Fig. 7B). In SLB-1 cells, HBZ and Bach2 were partially colocalized in the cytoplasm (Fig. 7B, white arrow and Supplementary Fig. 5B).

## Discussion

Chronic viral infections have been reported to be closely associated with various diseases due to persistent cytokine signaling, generation of reactive oxygen species (ROS), induction of DNA damage, and accelerated immune senescence [24]. In HTLV-1 infection, viral regulatory factor Tax has been reported to cause ROS production [25], transcriptional regulation of cytokine/chemokine genes via the NF-κB pathway [26], cell cycle regulation [27–29], and DNA damage response [30, 31] to HTLV-1 infected T cells. Collectively, these findings suggest that prolonged antigen stimulation by HTLV-1 infection alters the expression and regulation of genes involved in intracellular signaling pathways, such as the Menin-Bach2 pathway, which may contribute to the dysregulation of cytokine production associated with CD4 + T cell senescence.

Our data showed that the majority of HTLV-1-infected human leukemic T-cell lines examined (five out of eight) did not express Bach2 protein, but its expression was confirmed in all three HTLV1-negative human leukemic T-cell lines examined. In HTLV-1-infected cell lines (MT1, MT4, HUT102), which did not exhibit Bach2 protein expression, treatment with 5-Aza-dC induced Bach2 protein expression, suggesting that Bach2 protein expression may be suppressed through epigenetic mechanisms in these cells. In contrast, this phenomenon was not observed in two out of three HTLV-1-uninfected cell lines (Jurkat, Molt4). Furthermore, using JPX-9, which allows for the induction of Tax with metal ions, we confirmed that the expression of Bach2 is enhanced along with Tax expression. This suggests the possibility that dynamics of Bach2 may be closely related to immune evasion, survival of HTLV-1-infected T cells in vivo, and the oncogenic mechanisms of ATL. Meanwhile, our data showed a statistically significant reduction in the expression level of *menin* mRNA in PBMCs from HAM/TSP and ATL patients compared to NCs, and the expression level of *bach2* mRNA in PBMCs from HTLV-1 infected individuals was significantly lower than that in NCs, irrespective of the infection status (i.e., HAM/TSP, ATL, ACs). These results indicate that both HTLV-1-infected cell lines and PBMCs from infected individuals show dysregulation of the Menin-Bach2 pathway. Since Bach2 supports the generation of Treg cells and suppresses effector T cells to prevent the onset of autoimmune disease and chronic inflammation [17, 18], suppression of Bach2 expression in HTLV-1-infected individuals may be one strategy by which HTLV-1 evades host immune responses and maintains chronic infection. Also, previous reports have shown that downregulation of Bach2 expression in CD4 + T cells leads to dysregulation of cytokine production and cellular immune responses in vivo [14, 17–20], which is a characteristic feature of the inflammatory disease HAM/TSP and is an important finding that contributes to our understanding of the pathogenesis of HAM/TSP.

Co-immunoprecipitation (co-IP) experiments demonstrated that both Menin and its molecular target Bach2 directly interact with HTLV-1 Tax and HBZ, respectively, suggesting that both Tax and HBZ may be involved in cellular processes associated with the Menin-Bach2 pathway. However, the interaction may be weak or transient, as endogenous Bach2 protein could not be detected by co-IP with Tax or HBZ in MT-2 cells. The importance of the Menin-Bach2 pathway in HTLV-1 infection is further supported by the fact that an inhibitor of the Menin-MLL interaction, named MI-2-2, inhibited the proliferation of HTLV-1 transformed T-cell line MT-2, but not SLB-1. In general, the differences in drug effects across cell lines can be attributed to various factors, such as differences in the expression of molecular targets, variations in signaling pathways, differences in drug uptake and efflux, and alterations in apoptotic pathways. Thus, the opposing effects on cell proliferation in these T cell lines suggest that the proliferation of HTLV-1 infected cells is controlled through at least two independent mechanisms mediated by Tax and/or HBZ via the Menin-Bach2 pathway. This also suggests that the inhibition of the Menin-MLL interaction may represent a candidate therapeutic approach for some ATL patients. Regarding the opposite effects of MI-2-2 on MT2 and SLB1, the differences in the interaction of Bach2 with the viral transcriptional regulators Tax or HBZ are of particular interest.

To further explore the causes of the opposing effects of the Menin-MLL interaction inhibitors, we examined the effects of *tax* or *HBZ*-directed shRNAs in both SLB-1 and MT-2 cells. In both MT-2 and SLB-1 cells, the knockdown of HTLV-1 Tax resulted in a decrease in Bach2 but not Menin, whereas the knockdown of HBZ did not change the expression levels of either Menin and Bach2. The knockdown of Tax and HBZ was independent of the inhibition of cell proliferation by the Menin-MLL interaction inhibitor MI-2-2. In contrast, the knockdown of Menin by siRNA resulted in a decrease in Bach2 in MT-2 cells, in which cell growth was suppressed by the Menin-MLL interaction inhibitor, whereas the knockdown of Menin did not change the expression levels of Bach2 protein in SLB-1 cells, in which cell growth was not affected by the Menin-MLL inhibitor MI-2-2. Thus, the decreased expression of Bach2 led by Menin knockdown was associated with the inhibition of cell proliferation by the Menin-MLL interaction inhibitor MI-2-2. In other words, MI-2-2 is no longer effective in cells in which the Menin-Bach2 pathway does not function properly.

We further revealed the different subcellular localization patterns of HBZ and Menin between MT-2 and SLB-1 cells using indirect immunofluorescence staining. Tax and Bach2 were localized in the cytoplasm in both MT-2 and SLB-1 cells, whereas HBZ and Menin localized predominantly in the nucleus in MT-2 cells but predominantly localized in the cytoplasm in SLB-1 cells, and in SLB-1 cells, HBZ and Bach2 were partially co-localized in the cytoplasm. We found that the predominant localization of HBZ and Menin in the nucleus, which was observed in MT-2 cells, was associated with the proper function of the Menin-Bach2 pathway. Knockdown of Menin downregulated Bach2 expression in MT-2 but not in SLB-1 cells, and inhibition of the Menin-MLL interaction inhibited the growth of MT-2 but not SLB-1 cells. Interestingly, previous studies have shown that HBZ impedes the tumor-suppressive activity of Menin and promotes JunD-mediated leukemogenesis [32]. This suggests that in HTLV-1 infection, HBZ, which is differentially localized in MT-2 and SLB-1 cells, may act as a leukemogenic stimulator that inhibits the tumor-suppressive activity of Menin and promotes JunD-mediated leukemogenesis.

In addition to neurological symptoms, some HAM/TSP patients also exhibit autoimmune-like disorders, such as uveitis, arthritis, T-lymphocyte alveolitis, polymyositis, and Sjögren syndrome [9]. Multiple sclerosis (MS) is a chronic autoimmune inflammatory disease of the central nervous system characterized pathologically by demyelination, gliosis, neuroaxonal damage and inflammation, and especially, the primary progressive form of MS (PPMS) is clinically, pathologically and immunologically similar to HAM/TSP. Both MS and HAM/TSP are caused by complex interactions among multiple genes and environmental factors. Most importantly, recent genome-wide association studies [33, 34] and gene expression analyses [35] revealed that *bach2* gene is a new susceptibility gene for MS. Interestingly, *bach2* was downregulated in the blood cells of MS patients compared to healthy subjects, as observed in HAM/TSP patients in our present study. Furthermore, in infections caused by other human retrovirus HIV-1, strong integration clusters in *bach2* have been observed in clonally expanded latently infected cells [36–40]. the expression of Bach2 was downregulated in patients with the Epstein–Barr virus (EBV)-positive subtype of diffuse large B-cell lymphoma (DLBCL) [41]. These observations suggest that certain chronic viral infections are associated with *bach2* gene. Similarly, it is possible that the decreased expression of *bach2* in the PBMCs of HTLV-1-infected individuals, including HAM/TSP and ATL patients, is associated with the development of these diseases and prolonged latent virus infection via the functions of the viral regulators Tax and/or HBZ. Dysregulation of the Menin-Bach2 pathway, particularly Bach2 downregulation, is assumed to result from alterations in various cellular processes, including protein degradation and epigenetic mechanisms. Further studies are needed to define the in vivo significance and its participation in the pathogenesis and progression of HTLV-1 associated diseases such as HAM/TSP and ATL.

## Conclusion

In this study, we demonstrated that HTLV-1 infection alters the regulation of the Menin-Bach2 pathway, which controls cell proliferation. The Menin-MLL interaction inhibitor loses its effectiveness in suppressing cell proliferation when Menin loses control over Bach2 expression. Dysregulation of the Menin-Bach2 pathway may contribute to the pathogenesis of HTLV-1 associated cancer and inflammatory diseases via mechanisms mediated by Tax and/or HBZ.

## Methods

### Study population and preparation of clinical samples

This study was approved by the Research Ethics Committee of the Kawasaki Medical School (approval number: 3447-01). The study was explained to all participants orally and in writing, and written informed consent was obtained. All procedures were performed in accordance with the national guidelines for clinical research in Japan (i.e., the Ethical Guidelines for Medical and Health Research Involving Human Subjects) and the Declaration of Helsinki. The characteristics of HTLV-1-infected and -uninfected study participants are summarized in Table 1. We investigated HTLV-1 proviral load (PVL) and the mRNA expression of *tax*, *HBZ*, *menin* and *bach2* in HTLV-1 infected individuals as follows: 21 patients with ATL (acute-type, *n* = 12; lymphoma-type, *n* = 6; smoldering-type, *n* = 2; chronic-type, *n* = 1), 13 patients with HAM/TSP, 11 ACs, and 11 NCs. These samples were chosen randomly. Diagnoses of HAM/TSP and ATL were based on the World Health Organization diagnostic criteria [42] and Shimoyama criteria [43], respectively. Fresh PBMCs were isolated using Histopaque-1077 (Sigma, St. Louis, MO, USA) density gradient centrifugation, washed twice in RPMI 1640 medium, and stored in liquid nitrogen as stock lymphocytes until use.

**Table 1:** Characteristics of HTLV-1-infected and -uninfected study participants

	NCs (*n* = 11)	ACs (*n* = 11)	HAM/TSP (*n* = 13)	ATL (*n* = 21)
Age	52.1 ± 16.	54.3 ± 17.4	62.7 ± 11.7	65.4 ± 16.7
Sex, n (%)				
Male	6 (54.5)	5 (45.5)	4 (30.8)	11 (52.4)
Female	5 (45.5)	6 (54.5)	9 (69.2)	10 (47.6)
^a^ HTLV-1 proviral load (Median)	N/A	479.6 ± 441.6 (423.5)	1826.8 ± 877.0 (1442.0)	7850.8 ± 5877.8 (7286.0)

NCs: normal uninfected controls

ACs: asymptomatic HTLV-1 carriers

HAM/TSP: HTLV-1 associated myelopathy/tropical spastic paraparesis

ATL: adult T-cell leukemia/lymphoma

The results represent the mean ± SD

^a^ HTLV-1 tax copy number per 10^4^ PBMCs

N/A: not applicable

### Cells

Eight HTLV-1-infected human T-cell lines (MT1 [44], MT2 [45], MT4 [46], HUT102 [47], ATL43Tb [48], ED [49], C5MJ [50], and SLB1 [51]) and three HTLV-1-uninfected T-cell lines (CEM [52], Molt4 [52], and Jurkat [53]) were used in this study. These T cell lines were kindly provided by the collaborators (see the Acknowledgements section) or obtained from the Riken BioResource Research Center Cell Bank (Tsukuba, Japan). MT-2, MT-4, SLB-1, and C5/MJ are chronically HTLV-1-infected cell lines derived from cord blood mononuclear cells that were exposed to HTLV-1 isolated from patients with ATL, that is HTLV-1-transformed T cell lines. MT-1, HUT102, ATL43Tb, and ED are HTLV-1-infected cell lines derived from ATL patients. We also used the JPX9 cell line, a Jurkat subclone generated by the stable introduction of a functional Tax expression-plasmid vector. After adding CdCl_2_ to the culture medium (final concentration: 10 µM), JPX9 cells express biologically active Tax protein under the control of the metallothionein promoter [21]. Cells were cultured in RPMI 1640 medium supplemented with 10% heat-inactivated fetal calf serum (FCS), 50 U/ml penicillin, and 50 µg/ml streptomycin (Wako Chemicals, Osaka, Japan) at 37 °C in 5% CO_2_. HEK293T cells were grown in Dulbecco’s modified Eagle’s medium (DMEM) supplemented with 4 mM glutamine, 10% FCS, 100 units/ml penicillin, and 100 µg/ml streptomycin at 37 °C and 5% CO_2_.

### 5-aza-2’-deoxycytidine (5-aza-dC) treatment

HTLV-1 infected (MT1, MT4 and HUT102) or non-infected (Jurkat, CEM and Molt-4) human T-cell lines were seeded in 100-mm dishes at a density of 1 × 10^6^ cells per dish 1 day before drug treatment. The cells were treated with 10 µM 5-aza-dC (Sigma-Aldrich, St. Louis, MO) every 24 h for 3 days and then harvested for Western blotting.

### Western blotting

Whole cell lysates were extracted from human T cell lines using Pierce RIPA Buffer (Thermo Fisher Scientific, Waltham, MA, USA) with a protease inhibitor cocktail (Thermo Fisher Scientific). Briefly, cells were washed three times with PBS, resuspended in Pierce RIPA Buffer with protease inhibitor cocktail, and then sonicated on ice using a Bioruptor^®^ sonicator (Diagenode, Liège, Belgium), according to the manufacturer’s instructions. After centrifugation (14,000 × g, 4 °C, 15 min), supernatants were collected and subjected to SDS-polyacrylamide gel electrophoresis (SDS-PAGE) followed by transfer to polyvinylidene difluoride (PVDF) membranes (pore size 0.45 μm, Merck Millipore, MA, USA) for western blotting. PVDF membranes were blocked with 5% skim milk in Tris-buffered saline containing 0.1% Tween 20 (TBS-T), and probed with anti-Tax mouse monoclonal (clone Lt-4, mouse IgG3) [54], anti-HBZ mouse monoclonal (clone #7 − 1, mouse IgG2b) [55], anti-Menin rabbit polyclonal (A300-105 A, Bethyl Laboratories, TX, USA), anti-Menin rabbit monoclonal (clone D45B1, Cell Signaling Technology, MA, USA), anti-Bach2 rabbit monoclonal (clone D3T3G, Cell Signaling Technology, MA, USA), anti-β-actin mouse monoclonal (sc-8432, Santa Cruz Biotechnology, TX, USA) or anti-a-Tubulin rabbit polyclonal (PM054, MBL, Tokyo, Japan) antibodies. The membranes were washed with TBS-T and incubated with IRDye 680RD goat anti-mouse IgG or IRDye 800CW goat anti-rabbit IgG (LI-COR Biosciences, Lincoln, NE, USA). After washing with TBS-T, the protein levels were assayed using the Odyssey CLx Infrared Imaging System (LI-COR Biosciences).

### RNA extraction and cDNA synthesis

RNA was extracted from the PBMCs using an RNeasy Mini Kit with on-column DNase digestion (Qiagen, Hilden, Germany). cDNA was synthesized using the PrimeScript^®^ RT Reagent Kit (Takara, Kyoto, Japan). All reactions were performed according to the manufacturer’s instructions.

### Quantification of HTLV-1 proviral load

To examine the HTLV-1 PVL, quantitative PCR (qPCR) using primers and probes for the most conserved HTLV-1 *tax* region (amplicon length: 223 bp) was performed using 100 ng of genomic DNA (roughly equivalent to 10^4^ cells) extracted from PBMCs as previously reported [56]. Based on the standard curve created by four known concentrations of template, the concentration of unknown samples can be determined. The amount of HTLV-1 proviral DNA was determined using the following formula: copy number of HTLV-1 *tax* per 1 × 10^4^ PBMCs = [(copy number of Tax)/(copy number of β − actin/2)] × 10^4^. All samples were examined in triplicate.

### Real-time quantitative reverse transcription PCR analysis

To estimate *menin* and *bach2* mRNA expression levels, qRT-PCR was performed using gene-specific primers (Hs 00365720_m1 for *menin* and Hs00222364_m1 for *bach2*; Applied Biosystems, Foster City, CA, USA). Expression levels of these genes were normalized to those of human hypoxanthine phosphoribosyltransferase 1 (HPRT1) (Human HPRT1 Endogenous Control 4333768; Applied Biosystems). All assays were performed in triplicates.

### Plasmid construction

The primers used for plasmid construction are listed in Table 2. To construct pCMV-HA-MENIN and pCMV-HA-BACH2, the full-length *menin* or *bach2* coding sequences were amplified by PCR using MEN1-FOR and MEN1-REV for *menin*, BACH2-FOR and BACH2-REV for *bach2* as primers, and the cDNA derived from MT-1 for *menin* or MT-2 cells for *bach2* as a template. The PCR products were digested with EcoRI and NotI, and cloned into EcoRI- and NotI-digested pCMV-HA-N (Clontech, CA, USA). To construct pCMV-FLAG-MENIN and pCMV-FLAG-BACH2, a FLAG-tagged fragment was generated by PCR using FLAG-FOR and FLAG-REV as primers without templates. The PCR products (i.e., FLAG-tag fragment) were digested with ApaI and EcoRI and cloned into ApaI- and NotI-digested pCMV-HA (i.e., the HA tag was removed by ApaI digestion) with EcoRI- and NotI-treated full-length *menin* or *bach2* fragments used for pCMV-HA-MENIN and pCMV-HA-BACH2 construction. The sequences of all recombinant plasmids were verified by Sanger sequencing. Expression vectors for Tax-FLAG (pCAGGS-P7-Tax-A-FLAGx2) [57] and HA-HBZ (pCMV-HA-HBZ) [55] been previously described.

**Table 2:** Primer sequences for plasmid construction

Primer name	Direction	Sequences (5’ to 3’)
MEN1-FOR	Forward	ATTAT **GAATTC** tg ggc ggc ATG GGG CTG AAG GCC GCC CAG AAG AC
MEN1-REV	Reverse	ATTAT **GCGGCCGC** ta TCA GAG GCC TTT GCG CTG CCG CTT GAG
FRAG-FOR	Forward	ATTAT **GGGCCC** acc ATG GAC TAC AAA GAC GAT GAC
FRAG-REV	Reverse	ATTAT **GAATTC** c CTT GTC GTC ATC GTC TTT GTA GTC
BACH2-FOR	Forward	ATTAT **GAATTC** tg ggc ggc ATG TCT GTG GAT GAG AAG CCT GAC TC
BACH2-REV	Reverse	ATTAT **GCGGCCGC** tca CTA GGT ATA ATC TTT CCT GGG CTG

GAATTC, GCGGCCGC, and GGGCCC sequences are the respective restriction sites for EcoRI, NotI, and ApaI

Underlines indicate the FLAG-tag sequences

### Immunoprecipitation assay

For the co-immunoprecipitation assay, 2.0 × 10^6^ HEK293T cells were plated in 6 cm-dishes in DMEM supplemented with 10% FCS 24 h before transfection. After exchange to flesh DMEM containing 10% FCS, cells were transfected with 1 µg of each indicated plasmid, i.e., pCAGGS-P7-Tax-A-FLAG, pCMV-HA-HBZ, pCMV-HA-MENIN, pCMV-HA-BACH2, pCMV-FLAG-MENIN, and pCMV-FLAG-BACH2 or empty vectors using Lipofectamine LTX (Invitrogen, Carlsbad, CA) according to the manufacturer’s instructions, and then cultured for 24 h at 37 °C. HTLV-1 infected MT2 cells (2.0 × 10^6^ cells/assay) were also used for the analysis of endogenous protein-protein interactions. The harvested cells were solubilized in lysis buffer (20 mM Tris-HCl [pH 7.9], 100 mM NaCl, and 0.1% Triton X-100). After sonication, homogenates were centrifuged at 14,000 × *g* at 4 °C for 5 min, and the supernatant fraction was used as extracts for immunoprecipitation. The cell extracts were incubated with ANTI-FLAG M2 Affinity Gel (SIGMA-ALDRICH Japan, Tokyo, Japan) or Protein A Sepharose conjugated with anti-HBZ antibody (clone #7 − 1) at 4 °C for 1 h. After incubation, the resins were collected by brief centrifugation and washed twice with lysis buffer. The resin-bound proteins were eluted by boiling in the SDS-PAGE Sample Loading Buffer (Takara Bio, Shiga, Japan) and subjected to 10% SDS-PAGE, followed by western blotting using anti-FLAG M2, anti-HBZ (clone #7 − 1), anti-Menin (A300-105 A), or anti-Bach2 (D3T3G) antibodies. An aliquot of the cell lysate removed before immunoprecipitation was used as an input (Fig. 3).

### Lentiviral transduction of short hairpin RNA (shRNA)

For lentivirus production, 5.0 × 10^5^ HEK293T cells were plated in a 6 cm-dish 24 h before transfection. Cells were transfected with 0.5 µg of lentiviral vectors expressing shRNA against Tax (pLKO.1-EGFP-shTax#1-EGFP and pLKO.1-EGFP-shTax#4-EGFP) or HBZ (pCSII-siHBZ31-EGFP), which were kindly provided by Professor Masao Matsuoka of Kumamoto University, or control shRNA (pLKO.1-negative shRNA-EGFP, Sigma-Aldrich) together with 5µl of Mission Lentiviral Packaging Mix (Sigma-Aldrich), which containing vesicular stomatitis virus G protein and the minimal set of lentiviral genes required to generate the virion structural protein and packaging functions, by using Lipofectamine LTX (Invitrogen) according to the manufacturer’s instructions. The culture supernatants were collected 48 h after transfection and filtered. HTLV-1-infected MT-2 and SLB-1 cells were infected with these lentiviruses for 16 h in the presence of 4 µg/ml polybrene or remained uninfected as a control of parental cells. Infected and uninfected cells were suspended in flesh RPMI1640 medium containing 10% FCS and incubated for 48 h. After 48 h incubation, infected cells were cultured in RPMI1640 containing 10% FCS in the presence of 1 µg/ml puromycin for an additional 48 h, other than the control of parental cells. These two infected and control parental cells were subjected to western blotting. The two infectants and control parental cells were also subjected to a trypan blue exclusion assay for cell growth analysis. The number of viable cells was determined daily by counting trypan blue-excluding cells using a hemocytometer.

### Treatment with *menin* small interfering RNAs

Stealth siRNAs (set of three; HSS106462, HSS106463, and HSS181079) were used to suppress *menin* gene expression. Stealth RNAi siRNA Negative Control Medium GC Duplex was used as the negative control. All reagents were obtained from Thermo Fisher Scientific. The 20 µM of each siRNAs were transfected into 5.0 × 10^6^ MT-2 or SLB-1 cells with the Neon™ Transfection System (Invitrogen). The cells were cultured in RPMI1640 medium containing 10% FCS for 48 h. After harvesting, the cell lysates were subjected to western blotting.

### Indirect immunofluorescence

MT-2 and SLB-1 cells were co-transfected with different combinations of expression plasmids. Cells were harvested 24 hrs after transfection, washed three times with PBS, and fixed in 100% ethanol for 5 min at -20°C. Fixed cells were washed with wash buffer (PBS containing 0.1% sodium azide and 0.1% BSA), and then incubated with anti-Tax (Lt-4), anti-HBZ (#7 − 1), anti-Menin (A300-105A), or anti-Bach2 (D3T3G) mAbs for 20 min at 4°C. After washing with wash buffer, cells were incubated with Alexa Fluor 488-conjugated secondary antibodies (goat anti-mouse IgG or goat anti-rat IgG; Cell Signaling Technology). The nuclei of the cells were stained blue with 4’,6-diamidino-2-phenylindole (DAPI). The cells were washed with PBS and mounted with 20% glycerol (Merck). Slides were examined using an LSM700 scanning laser confocal microscope (Carl Zeiss, Oberkochen, Germany).

### Statistical analysis

To test for significant differences among the four groups (HAM/TSP, ATL, ACs, and NCs), data were statistically analyzed using one-way analysis of variance (ANOVA). Intergroup comparisons were performed using Scheffé’s post-hoc multiple comparison test. The Mann–Whitney U test or Welch’s t-test was used to compare data between the two groups. The results shown represent the mean ± SD where applicable. The results were considered statistically significant at p values < 0.05.

## Electronic supplementary material

Below is the link to the electronic supplementary material.

Supplementary Material 1: **Fig. 1**. Effect of 5-aza-2’-deoxycytidine (5-aza-dC) treatment in HTLV-1-infected and non-infected T-cell lines. HTLV-1 infected (MT1, MT4 and HUT102) or non-infected (Jurkat, CEM and Molt-4) human T-cell lines were seeded in 100-mm dishes at a density of 1 × 10^6^ cells per dish 1 day before drug treatment. The cells were treated with 10 µM 5-aza-dC (Sigma-Aldrich, St. Louis, MO) every 24 h for 3 days and then harvested for Western blotting. (A) Two out of three HTLV-1 non-infected T-cell lines (Jurkat and Molt4) did not express Bach2 after 5-aza-dC treatment. (B) All HTLV-1-infected T-cell lines tested (MT1, MT4 and HUT102) expressed Bach2 after 5-aza-dC treatment.

Supplementary Material 2: **Fig. 2**. The expression of Bach2 is enhanced along with Tax expression. The JPX9 cell line is a Jurkat subclone generated by the stable introduction of a functional Tax expression-plasmid vector. After adding CdCl_2_ to the culture medium (final concentration: 10 µM), JPX9 cells express biologically active Tax protein under the control of the metallothionein promoter. (A) After adding CdCl_2_ to the culture medium (final concentration: 10 µM), JPX9 cells express Tax protein, and the expression of Bach2 is enhanced along with Tax expression. (B) Expression of *bach2* mRNA in PBMCs from HTLV-1 infected individuals is induced after adding CdCl_2_ to the culture medium (final concentration: 10 µM).

Supplementary Material 3: **Fig. 3**. The co-immunoprecipitation (co-IP) experiment using the HTLV-1 infected MT2 cell line. HTLV-1 infected MT2 cells were also used for the analysis of endogenous protein-protein interactions. (A) The interaction between endogenous Tax and Menin or Bach2 could not be detected by co-IP in MT-2 cells. (B) The interaction between endogenous HBZ and Menin or Bach2 could not be detected by co-IP in MT-2 cells.

Supplementary Material 4: **Fig. 4**. The effect of Menin-MLL interaction inhibitor in HTLV-1-infected and non-infected T-cell lines. The effect of the Menin-MLL inhibitor MI-2-2 on the proliferation of HTLV-1-infected and non-infected T cell lines was examined. The average of two independent experiments is shown. (A) The effect of the Menin-MLL inhibitor MI-2-2 on the proliferation of HTLV-1 infected T-cell lines (MT1, MT4, HUT102 and C5MJ). (B) The effect of the Menin-MLL inhibitor MI-2-2 on the proliferation of HTLV-1 non-infected T cell lines (Jurkat, CEM and Molt4).

Supplementary Material 5: **Fig. 5**. Subcellular localization of viral regulatory proteins and Menin-Bach2 pathway members. The subcellular localization of Menin and Bach2 in MT2 and SLB1 cells was determined by indirect immunofluorescence staining and the localization pattern was determined. The number of cells showing each localization pattern was expressed as the percentage of the total cell number. (A) HBZ was predominantly localized in the nucleus of MT-2 cells. Menin was mainly localized in the nuclei of MT2 cells. Bach2 is localized in the cytoplasm of MT2 cells. HBZ is predominantly localized in the cytoplasm of SLB1 cells. Menin was predominantly localized in the cytoplasm of SLB1 cells. Bach2 is localized in the cytoplasm of SLB1 cells. (B) HBZ and Bach2 were partially co-localized in the cytoplasm of SLB1 cells.

## Data Availability

No datasets were generated or analysed during the current study.

## Abbreviations

HTLV-1: Human T-cell leukemia virus type-1
ATL: Adult T-cell leukemia
HAM/TSP: HTLV-1-associated myelopathy/tropical spastic paraparesis
ACs: Asymptomatic carriers
NCs: Normal uninfected controls
