# Supplementary figures and images for: Dysregulation of the tumor suppressor Menin and its target Bach2 in HTLV-1 infection
Source: Retrovirology. 2025 Mar 25;22:3. doi: 10.1186/s12977-025-00660-7 (PMC11934541; doi:10.1186/s12977-025-00660-7)

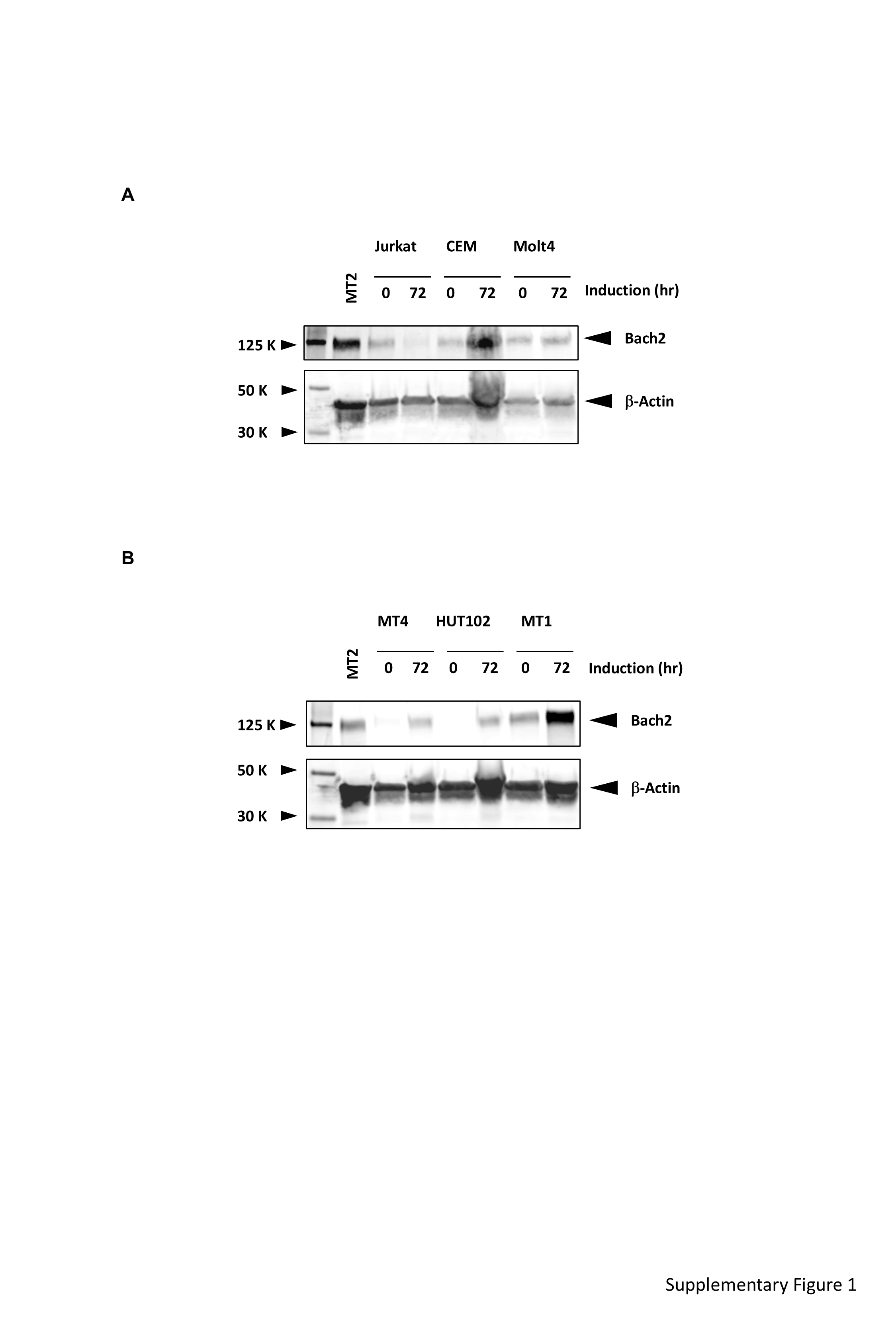

Supplement: Supplementary file 1 — Supplementary Material 1: Fig. 1. Effect of 5-aza-2’-deoxycytidine (5-aza-dC) treatment in HTLV-1-infected and non-infected T-cell lines. HTLV-1 infected (MT1, MT4 and HUT102) or non-infected (Jurkat, CEM and Molt-4) human T-cell lines were seeded in 100-mm dishes at a density of 1 × 10^6^ cells per dish 1 day before drug treatment. The cells were treated with 10 µM 5-aza-dC (Sigma-Aldrich, St. Louis, MO) every 24 h for 3 days and then harvested for Western blotting. (A) Two out of three HTLV-1 non-infected T-cell lines (Jurkat and Molt4) did not express Bach2 after 5-aza-dC treatment. (B) All HTLV-1-infected T-cell lines tested (MT1, MT4 and HUT102) expressed Bach2 after 5-aza-dC treatment. [file 12977_2025_660_MOESM1_ESM.tif]

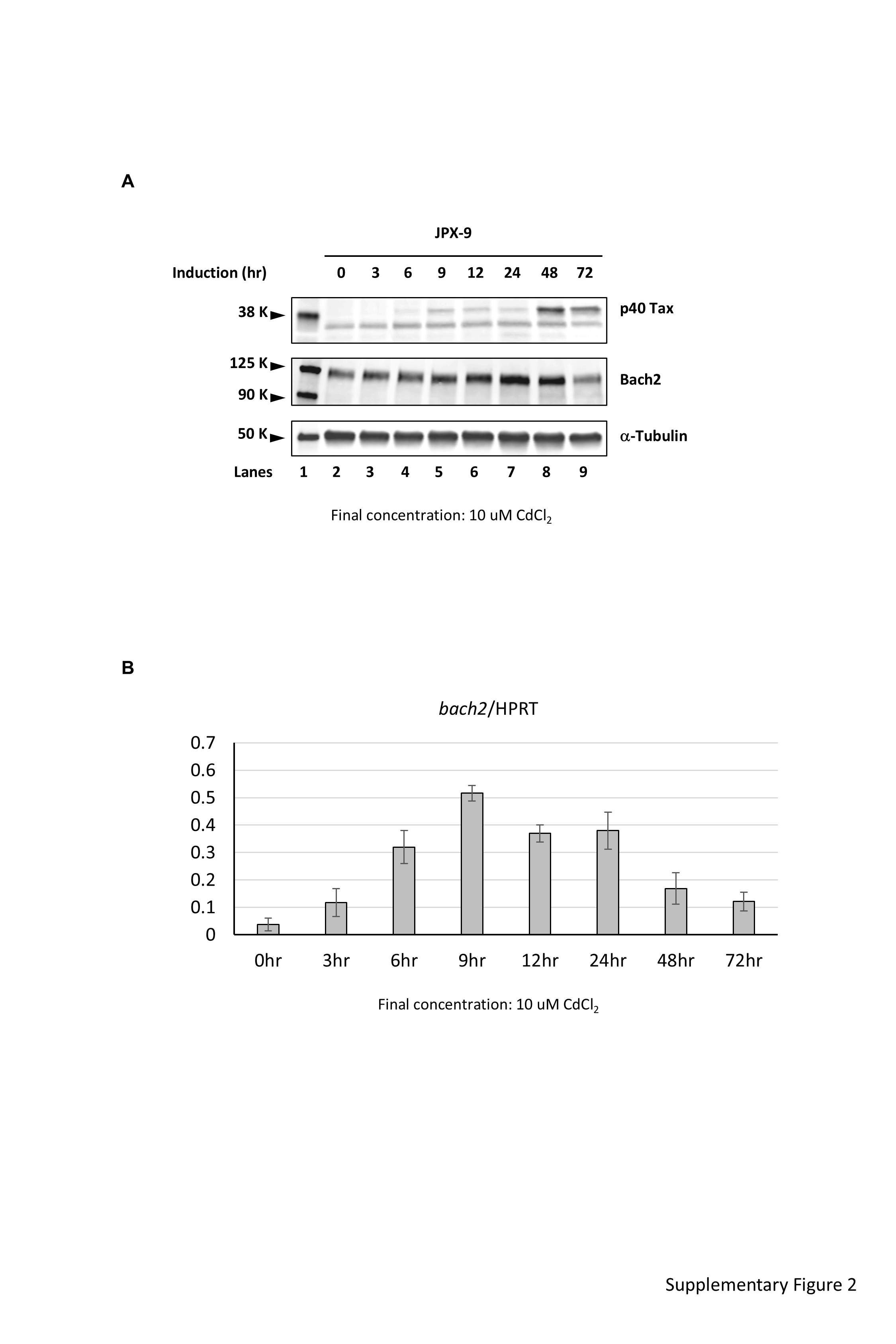

Supplement: Supplementary file 2 — Supplementary Material 2: Fig. 2. The expression of Bach2 is enhanced along with Tax expression. The JPX9 cell line is a Jurkat subclone generated by the stable introduction of a functional Tax expression-plasmid vector. After adding CdCl2 to the culture medium (final concentration: 10 µM), JPX9 cells express biologically active Tax protein under the control of the metallothionein promoter. (A) After adding CdCl2 to the culture medium (final concentration: 10 µM), JPX9 cells express Tax protein, and the expression of Bach2 is enhanced along with Tax expression. (B) Expression of bach2 mRNA in PBMCs from HTLV-1 infected individuals is induced after adding CdCl2 to the culture medium (final concentration: 10 µM). [file 12977_2025_660_MOESM2_ESM.tif]

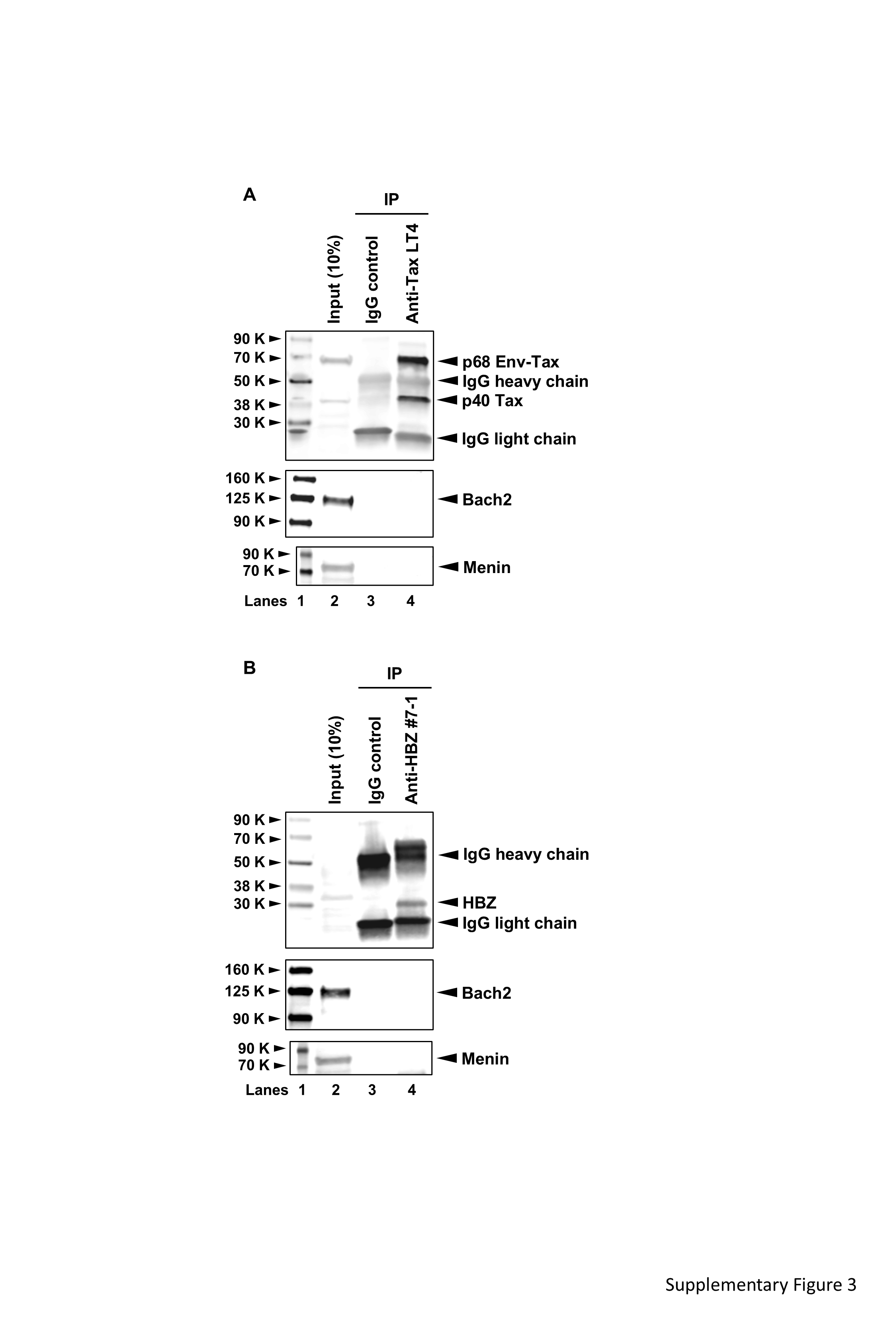

Supplement: Supplementary file 3 — Supplementary Material 3: Fig. 3. The co-immunoprecipitation (co-IP) experiment using the HTLV-1 infected MT2 cell line. HTLV-1 infected MT2 cells were also used for the analysis of endogenous protein-protein interactions. (A) The interaction between endogenous Tax and Menin or Bach2 could not be detected by co-IP in MT-2 cells. (B) The interaction between endogenous HBZ and Menin or Bach2 could not be detected by co-IP in MT-2 cells. [file 12977_2025_660_MOESM3_ESM.tif]

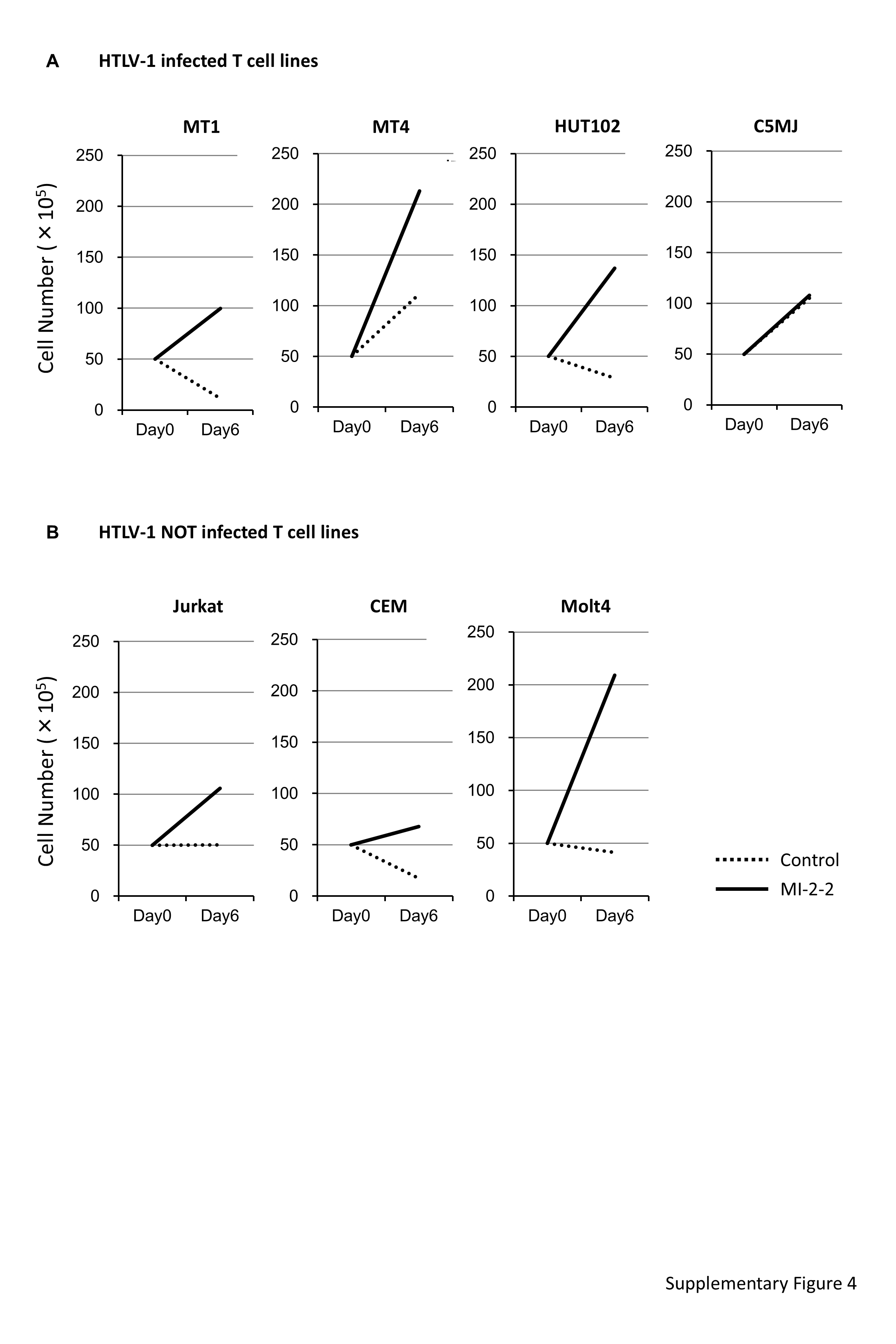

Supplement: Supplementary file 4 — Supplementary Material 4: Fig. 4. The effect of Menin-MLL interaction inhibitor in HTLV-1-infected and non-infected T-cell lines. The effect of the Menin-MLL inhibitor MI-2-2 on the proliferation of HTLV-1-infected and non-infected T cell lines was examined. The average of two independent experiments is shown. (A) The effect of the Menin-MLL inhibitor MI-2-2 on the proliferation of HTLV-1 infected T-cell lines (MT1, MT4, HUT102 and C5MJ). (B) The effect of the Menin-MLL inhibitor MI-2-2 on the proliferation of HTLV-1 non-infected T cell lines (Jurkat, CEM and Molt4). [file 12977_2025_660_MOESM4_ESM.tif]

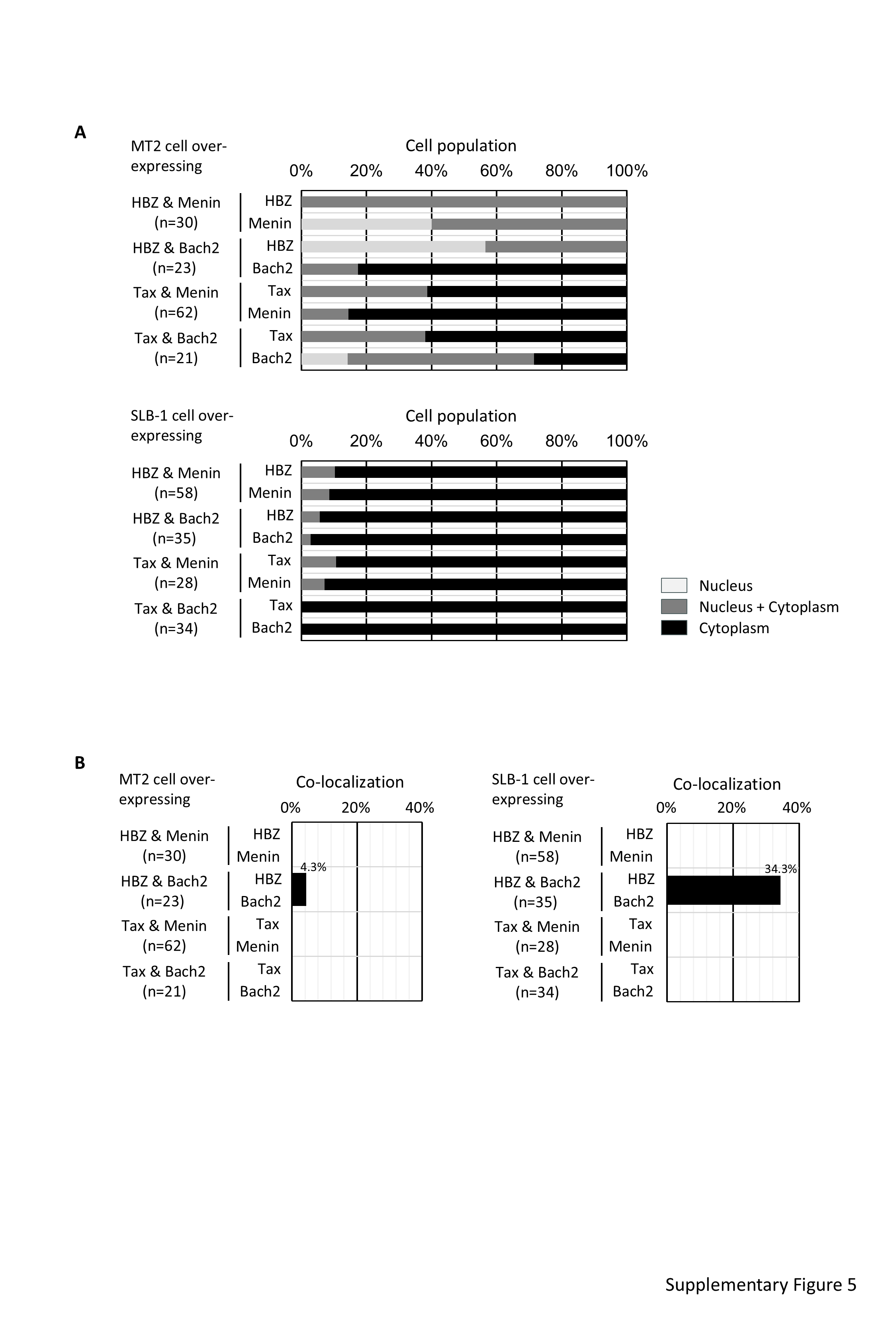

Supplement: Supplementary file 5 — Supplementary Material 5: Fig. 5. Subcellular localization of viral regulatory proteins and Menin-Bach2 pathway members. The subcellular localization of Menin and Bach2 in MT2 and SLB1 cells was determined by indirect immunofluorescence staining and the localization pattern was determined. The number of cells showing each localization pattern was expressed as the percentage of the total cell number. (A) HBZ was predominantly localized in the nucleus of MT-2 cells. Menin was mainly localized in the nuclei of MT2 cells. Bach2 is localized in the cytoplasm of MT2 cells. HBZ is predominantly localized in the cytoplasm of SLB1 cells. Menin was predominantly localized in the cytoplasm of SLB1 cells. Bach2 is localized in the cytoplasm of SLB1 cells. (B) HBZ and Bach2 were partially co-localized in the cytoplasm of SLB1 cells. [file 12977_2025_660_MOESM5_ESM.tif]
